# SARS-CoV-2 Switches ‘on’ MAPK and NFκB Signaling via the Reduction of Nuclear DUSP1 and DUSP5 Expression

**DOI:** 10.3389/fphar.2021.631879

**Published:** 2021-04-20

**Authors:** Swati Goel, Fatemeh Saheb Sharif-Askari, Narjes Saheb Sharif Askari, Bushra Madkhana, Ahmad Munzer Alwaa, Bassam Mahboub, Adel M Zakeri, Elaref Ratemi, Rifat Hamoudi, Qutayba Hamid, Rabih Halwani

**Affiliations:** ^1^ Sharjah Institute of Medical Research, University of Sharjah, Sharjah, United Arab Emirates; ^2^ Department of Clinical Sciences, College of Medicine, University of Sharjah, Sharjah, United Arab Emirates; ^3^ Rashid Hospital, Dubai Health Authority, Dubai, United Arab Emirates; ^4^ Department of Plant Production, Faculty of Agriculture and Food Sciences, King Saud University, Riyadh, Saudi Arabia; ^5^ Jubail- Industrial College, Department of Chemical and Process Engineering Technology, Jubail- Industrial City, Al Jubail, Saudi Arabia; ^6^ Meakins-Christie Laboratories, Research Institute of the McGill University Health Center, Montreal, QC, Canada; ^7^ Prince Abdullah Ben Khaled Celiac Disease Chair, Department of Pediatrics, Faculty of Medicine, King Saud University, Riyadh, Saudi Arabia

**Keywords:** SARS-CoV-2, COVID-19, MAPK, NFkB, DUSP1, DUSP5, chroloquine

## Abstract

Mitogen-activated protein kinases (MAPK) and NF-kappaB (NF-κB) pathway regulate many cellular processes and are essential for immune cells function. Their activity is controlled by dual-specificity phosphatases (*DUSPs*). A comprehensive analysis of publicly available gene expression data sets of human airway epithelial cells (AECs) infected with SARS-CoV-2 identified *DUSP1* and *DUSP5* among the lowest induced transcripts within these pathways. These proteins are known to downregulate MAPK and NF-κB pathways; and their lower expression was associated with increased activity of MAPK and NF-κB signaling and enhanced expression of proinflammatory cytokines such as *TNF-α*. Infection with other coronaviruses did not have a similar effect on these genes. Interestingly, treatment with chloroquine and/or non-steroidal anti-inflammatory drugs counteracted the SARS-CoV-2 induced reduction of *DUSP1* and *DUSP5* genes expression. Therapeutically, impeding this evasion mechanism of SARS-CoV-2 may help control the exaggerated activation of these immune regulatory pathways during a COVID-19 infection.

## Introduction

The proinflammatory cytokine storm, an unregulated amplification of pro-inflammatory cytokines, is one of the hallmarks of a severe coronavirus infection (COVID-19) (Costela-Ruiz et al., 2020; Haberman et al., 2020). The uncontrolled viral replication, due to low or delayed type I interferons production, and the resulting tissue damage, exaggerate the level of pro-inflammatory cytokines produced and result in a cytokine storm (Bastard et al., 2020; Zhang et al., 2020). The pro-inflammatory cytokines produced further attract immune cells and lead to a widespread lung inflammation (Hojyo et al., 2020). These uncontrolled inflammatory responses in patients with severe COVID-19 could lead to acute respiratory syndrome coronavirus 2 (SARS-CoV-2)-induced immune abnormalities characterized by lymphopenia, lymphocyte dysfunction, and granulocyte and monocyte abnormalities which may result in septic shock and multiple organ dysfunction (Yang et al., 2020). Therefore, the mechanisms underlying marked pro-inflammatory mediator release in patients with COVID-19 must be identified to guide the clinical management of the disease.

The activation of intracellular signaling pathways, such as mitogen-activated protein kinase (MAPK) and nuclear factor kappa B (NF-κB), are fundamental for cytokines production during a SARS-CoV-2 infection (Battagello et al., 2020). The increasing pro-inflammatory cytokine production through these pathways damages airway epithelial cells and alveolar tissues, resulting in decreased ventilation, acute lung injury, and acute respiratory distress syndrome (ARDS) (Puchelle et al., 2006; Qin et al., 2020; Song et al., 2020). Most of these mediators have pleiotropic downstream effects and are independent in their biological functions (Battagello et al., 2020).

MAPK is a main cell signaling pathway that is known to be activated by a wide variety of viruses (Kumar et al., 2018). In mammals, extracellular signal-regulated kinase (ERK), Janus kinase (JNK), and p38 MAPK are three major MAPK pathways. The p38 MAPK pathway mediates the cellular response to environmental stress and pathogenic infection. Previous reports of the related human coronavirus SARS-CoV-1 infection showed the activation of the p38 MAPK pathway and the enhancement of phosphorylation of its downstream regulated proteins, particularly kinases (Mizutani et al., 2004). Following the activation of kinases, downstream proteins of the pathway that include transcription factors and RNA binding proteins, promote the production of pro-inflammatory cytokine, such as tumour necrosis factor-alpha (*TNF-α*) (Cuadrado and Nebreda, 2010). An analysis of estimated transcription factor activity from gene expression data derived from SARS-CoV-2-infected primary human bronchial epithelial cells revealed that transcription factors regulated by the p38 MAPK were the most highly activated upon infection (Blanco-Melo et al., 2020a). Interestingly, the p38 MAPK inhibitor reduced the gene expression levels of *TNF-α* and other inflammatory cytokines that were increased during the SARS-CoV-2 infection of lung cancer cells in a dose-dependent manner, referring to p38 MAPK as a potential pharmacological target for COVID-19 (Bouhaddou et al., 2020).

The activation of NF-κB is a hallmark of many viral infections, and triggering of NF-κB activation is particularly relevant during infection with viruses that have NF-κB binding sites in their genome. Upon binding, the phosphorylation of IκBs by IκB kinases leads to the nuclear translocation of nuclear factors (NF) and the binding to their transcription factors which hence activate the transcription of a wide variety of pro-inflammatory mediators, such as *TNF-α* and *IL-8* (Santoro et al., 2003; Oeckinghaus and Ghosh, 2009). SARS-CoV-1 was shown to activate the NF-κB pathway (Liao et al., 2005), leading to the exaggerated expression of proinflammatory cytokines, and thus resulting in the production of a cytokine strom (Hirano and Murakami, 2020). Many reports have shown that NF-κB plays an important role in the pathogenesis of lung diseases during SARS-CoV-1 infection. The level of NF-κB expression was higher in the lungs of recombinant SARS-CoV-1-infected mice (DeDiego et al., 2014); while inhibitors of NF-κB improved survival of these mice and reduced SARS-CoV-1-induced inflammation. NF-κB is specifically induced by SARS-CoV-1 spike proteins to produce inflammatory mediators that are associated with ARDS (Dosch et al., 2009).

Similar to the Middle East Respiratory Syndrome coronavirus (MERS-CoV) and SARS-CoV-1, SARS-CoV-2 activates p38 MAPK and NF-κB pathways (Battagello et al., 2020). A selective inhibition of these pathways may hence attenuate the exaggerated inflammation observed during COVID-19 infection. Dual-specificity phosphatases (*DUSP*) genes are negative regulators of p38 MAPK signaling (Caunt and Keyse, 2013). *DUSP1* overexpression suppressed p38 MAPK signaling. Moreover, the silencing of *DUSP1* enhanced the pathway and induced proinflammatory cytokines such as *IL-6* and *IL-8*, in cells infected with the avian coronavirus infectious bronchitis virus (DeDiego et al., 2014). The overexpression of *DUSP5* negatively regulated both p38 MAPK and NF-κB signaling and attenuated the production of lipopolysaccharide (LPS)-mediated *TNF-α* and *IL-6*; whereas the knockdown of *DUSP5* increased their expression (Seo et al., 2017). Therefore, understanding the regulators of p38 MAPK and NF-κB activation may pave the way for targeting these molecules as a treatment strategy for COVID-19.

Here, we evaluated the expression levels of MAPKs and NFκB pathway-associated genes following the infection of primary human airway epithelial cells (AECs) with SARS-CoV-2, in comparison to SARS-CoV-1 and MERS-CoV infections. Defining the host response to SARS-CoV-2, as compared to other coronaviruses, is fundamental to identifying mechanisms of pathogenicity and potential therapeutic targets. Here, we found that modulator genes of these pathways (*DUSP1* and *DUSP5*) are expressed at a lower level in SARS-CoV-2 as compared to SARS-CoV-1 and MERS-CoV. Furthermore, the ability of anti-inflammatory medications to thwart the SARS-CoV-2 regulation of these pathways was assessed.

## Methods

### Gene Expression Datasets

In this study, bioinformatic analyses were conducted to investigate the differential expressions of MAPK and NF-κB pathway-associated genes following infection of AECs cells with either SARS-CoV-2, SARS-CoV-1 or MERS-CoV. Gene expression datasets deposited in the National Center for Biotechnology Information Gene Expression Omnibus (NCIB GEO, http://www.ncbi.nlm.nih.gov/geo), European Bioinformatics Institute (EMBL-EBI, https://www.ebi.ac.uk), and Toxicogenomics Project-Genomics Assisted Toxicity Evaluation System (Open TG-GATEs) (Igarashi et al., 2015) were used. The datasets of coronavirus infected human AECs included in the analysis are as follow; a dataset for SARS-CoV-2 infected AECs (GSE147507) (Blanco-Melo et al., 2020b), 3 datasets for SARS-CoV-1 infected AECs (GSE47960, GSE47961, and GSE47962) (Mitchell et al., 2013), and a dataset for MERS-CoV infected AECs (GSE81909). For all these datasets, the time point of 24 h post-infection was considered. Furthermore, we have used a dataset of COVID-19 nasopharyngeal swabs (GSE152075) which included 430 patients with COVID-19 and 54 healthy control individuals (Lieberman et al., 2020). For the animal studies, datasets of *DUSP1* deficient (GSE3565) (Hammer et al., 2006), and *DUSP5* deficient (GSE62999) (Holmes et al., 2015) mouse models were used. Spleen tissue from DUSP1-deficient mice (GSE3565) (Hammer et al., 2006) and bone marrow-derived eosinophils from DUSP5-deficient mice (GSE62999). Datasets of medication treatments were extracted from the TG-GATEs database and the high and middle doses were used, as previously described (Sharif-Askari et al., 2020a; Sharif-Askari et al., 2020b; Sharif-Askari et al., 2020c). The data for chloroquine treatment was extracted from the GSE30351 dataset. Details of the datasets used are presented in Supplementary Table S1. Furthermore, information on dose, duration and number of treatments are provided in Supplementary Table S2.

### Analysis of Trasncriptpomic Datasets

All the included raw data were evaluated for quality control. The raw Affymetrix data were normalized and log transformed. Microarray data (CEL files) were preprocessed with the Robust Multi-Array Average (RMA) technique (Hughey and Butte, 2015). Log-transformed, normalized intensities were used in Linear Models for Microarray (*LIMMA)* data analyses to identify differentially expressed genes between mock and SARS-CoV-1 infections (Dudoit et al., 2002; Smyth, 2004). RNA-sequencing data were processed with the *Limma-voom function*, which takes gene-level counts as its input, filters and normalizes the data, and through linear modelling, assesses differential expression and perform gene set testing (Ritchie et al., 2015).

**FIGURE 1:**
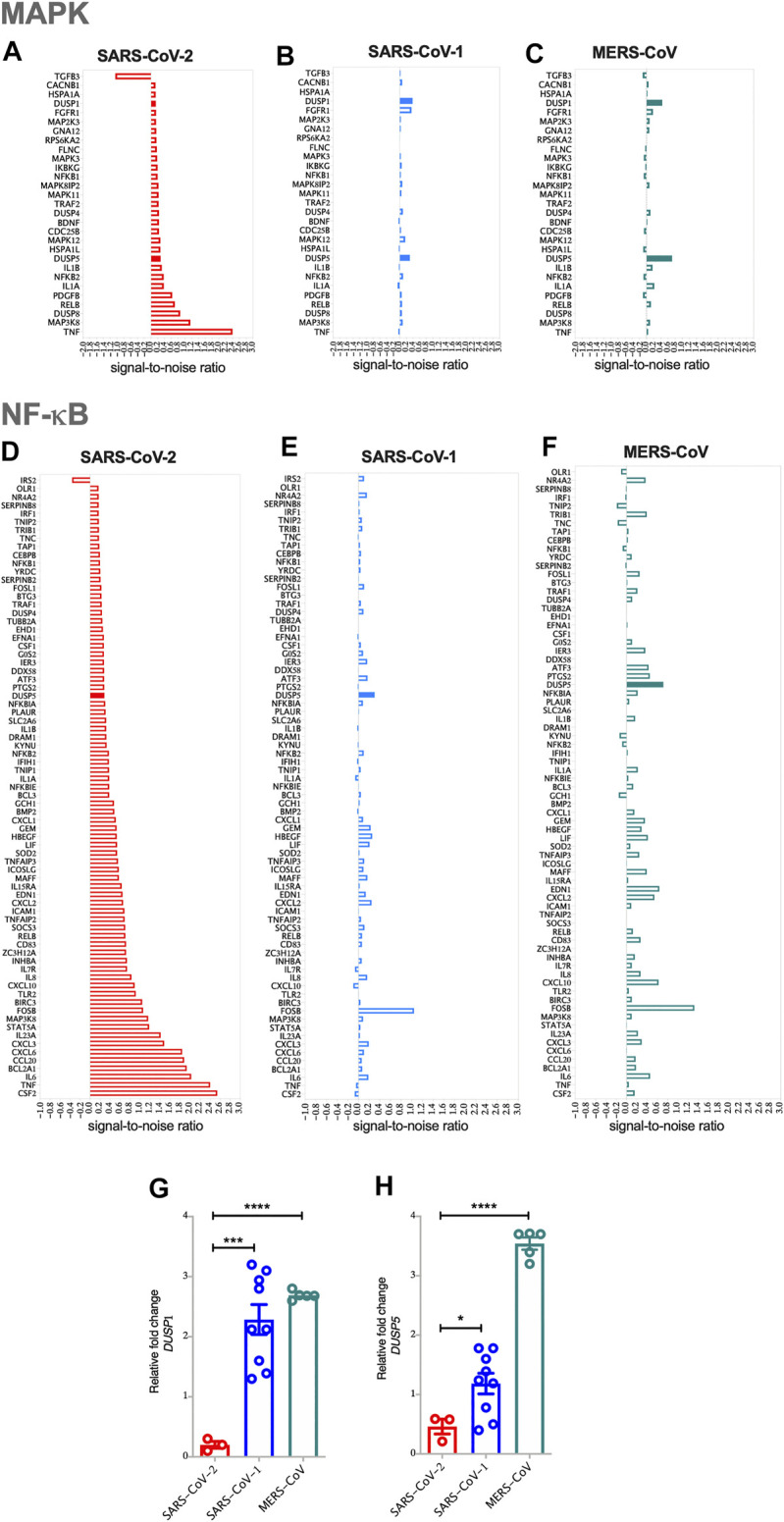
SARS-CoV-2 activates MAPK and NF-κB pathways via the reduced expression of *DUSP1* and *DUSP5* expression compared to other coronaviruses **(A)** Enrichment of MAPK pathway in SARS-CoV-2 infected human AECs along with the expression levels of enriched MAPK pathway related genes in SARS-CoV-2 infected AECs (ES = 0.437; NES = 1.57) **(B)** Enrichment of MAPK pathway in SARS-CoV-1 infected human AECs (ES = 0.489; NES = 1.51) **(C)** Enrichment of MAPK pathway in MERS-CoV infected human AECs (ES = 0.432; NES = 1.37) **(D)** Enrichment of NF-κB pathway in SARS-CoV-2 infected human AECs along with the expression levels of enriched NF-κB pathway related genes in SARS-CoV-2 infected AECs (ES = 0.663; NES = 1.24) **(E)** Enrichment of NF-κB pathway in SARS-CoV-1 infected human AECs (ES = 0.643; NES = 1.6) **(F)** Enrichment of NF-κB pathway in MERS-CoV infected human AECs (ES = 0.704; NES = 1.13). Enrichment Score (ES), Normalized enrichment Score (NES) **(G,H)** Gene expression of *DUSP1* and *DUSP5* in SARS-CoV-2, SARS-CoV-1, or MERS-CoV infected human AECs, 24 h post-infection. Representative data shows lower expression level of *DUSP1* and *DUSP5* following SARS-CoV-2 infection as compared to other coronaviruses. Two-way comparison was done using *t*-test or Mann-Whitney *U* test, depending on the skewness of the data. ***p < 0.001, ****p < 0.0001.

**FIGURE 2:**
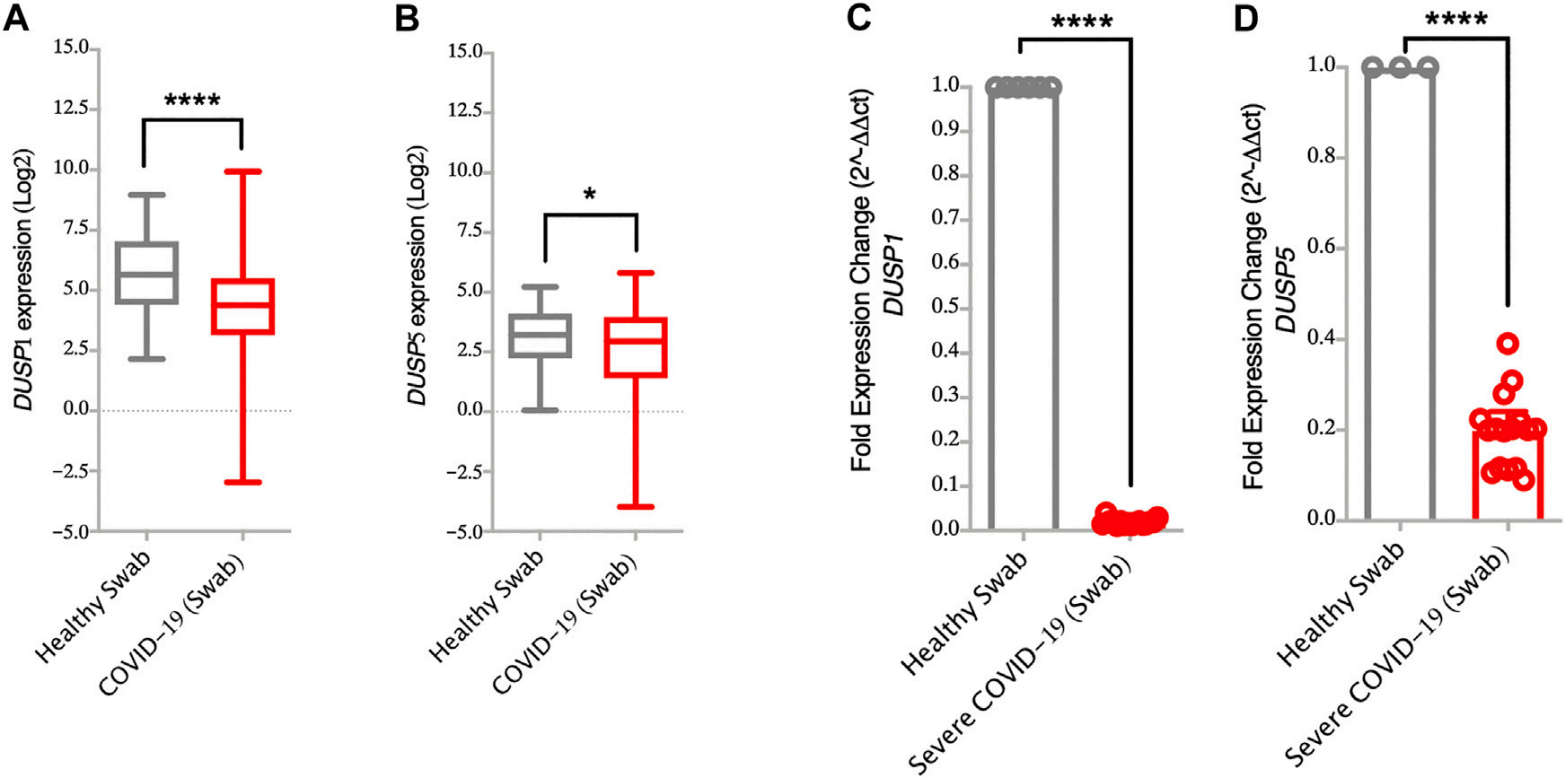
Lower gene expression levels of *DUSP1* and *DUSP5* in nasopharyngeal swabs from COVID-19 patients compared to healthy controls **(A,B)** Gene expression level of *DUSP1* and *DUSP5* in COVID-19 (*n* = 430) and in healthy (*n* = 54) nasopharyngeal swabs (Dataset GSE152075). Expression level of *DUSP1* and *DUSP5* is significantly lower in nasopharyngeal swabs from COVID-19 patients as compared to healthy controls **(C,D)** Confirmation of gene expression level of *DUSP1* and *DUSP5* as measured by RT-PCR, in nasopharyngeal swabs of severe COVID-19 patients (n=16) and healthy controls (n=6). Expression level of *DUSP1* and *DUSP5* is lower in nasopharyngeal swabs from COVID-19 patients as compared to healthy controls. Two-way comparison was done using *t*-test or Mann-Whitney *U* test, depending on the skewness of the data. *p < 0.05, ****p < 0.0001.

For the pathway analysis, expressed genes in each MAPK and NF-κB pathway were examined using Gene Set Enrichment Analysis (GSEA). This method first ranks the expression value of each gene by signal-to-noise ratio |S2N|- which is an estimate of the difference in gene expression means between two experimental settings. Then, it calculates the Enrichment Score (ES), by assessing the ranked list of genes in a given set (e.g., MAPK) using running-sum statistics. Moreover, it identifies the top or bottom leading-edge subsets that can be interpreted as the core of the given set accounting for the enrichment signals (Subramanian et al., 2005). The genes in a gene set (MAPK or NF-κB) are experimentally derived and are available at the Molecular signatures database (MsigDB) (Liberzon et al., 2011). The signal-to-noise ratio values of the genes in the enrichment core of MAPK or NF-κB set were plotted in Figures 1,2. All analyses were performed by using the R software, version 3.6.1. (The R Foundation; http://www.r-project.org/).

### RT-PCR

Nasopharyngeal swabs were isolated from 16 severe COVID-19 patients (average age of 48 ± 8 years), and 6 healthy individuals (average age of 45 ± 7 years). The Dubai Scientific Research Ethics Committee (DSREC) approved this study. Written, informed consents were obtained from all study participants prior to inclusion. Precautions recommended by CDC for safe collecting, handling and testing of biological fluids were followed. The total RNA was isolated using the Trizol reagent according to the manufacturer’s instructions (Invitrogen, Carlsbad, CA, United States). For cDNA amplification, 5x Hot FirePol EvaGreen qRT-PCR SuperMix (Solis Biodyne) was used and RT-qPCR was performed in QuantStudio 3 Real-Time PCR System (Applied Biosystems). Primer sequences for *DUSP1, DUSP5, TNF-α, IL-1β, IL-1A, IL-6, IL-8*, and *IL-23* used in qRT-PCR, are deposited in Supplementary Table S3. Gene expression was analyzed using the Comparative Ct (ΔΔCt) method upon normalization to the reference gene 18s rRNA (Kuchipudi et al., 2012). The data were checked for normal distribution using the Shapiro-wall test and depending on the skewness of the data student t-test or Mann-Whitney *U* test was carried out in GraphPad Prism version 8.4.1 (GraphPad Software, San Diego, Calif). For all analyses, *p*-values <0.05 were considered significant.

## Results

### DUSP1 and DUSP5 Gene Expression Are Reduced During SARS-CoV-2 Infection

The pathology of SARS-COV-2 is partly driven by a storm of cytokines, most of which are regulated by MAPK and NF-κB signaling patways. To evaluate the involvement of these two pathways, GSEA was carried out on SARS-CoV-2, SARS-CoV-1 or MERS-CoV infected AEC. Compared to SARS-CoV-1 and MERS-CoV, infection with SARS-CoV-2 resulted in higher MAPK and NF-κB pathway activation in AEC, apparent by the upregulation of the most common genes regulating these pathways (Figure 1). The expression levels of *TNF-α* and *MAP3K8* within the MAPK pathway; and *CSF2, TNF, IL-6, BCL2A1, CCL20, CXCL6, CXCL3,* and *IL-23* within the NF-κB pathway, were significantly elevated (Figures 1A,D).

Interestingly, infection with SARS-CoV-2 resulted in lower expression level of *DUSPs*, including *DUSP1* and *DUSP5* in infected AEC as compared to SARS-CoV-1 and MERS-CoV (Figures 1A–H). Lower gene expression levels of *DUSP1* and *DUSP5* was also observed in nasopharyngeal swabs from patients with SARS-CoV-2 infection (Figures 2A,B). To confirm this observation, we analyzed the expression of *DUSP1* and *DUSP5* in nasopharyngeal swabs from severe COVID-19 patients recruited from a local hospital. The level of these two genes were significantly lower in nasopharyngeal swabs of severe COVID-19 patients compared to healthy individuals (Figures 2C,D).

### Reduced Gene Expression of *DUSP1* Correlated With Lower *TNF-α, IL-1β* and *IL-1A* Cytokines

**FIGURE 3:**
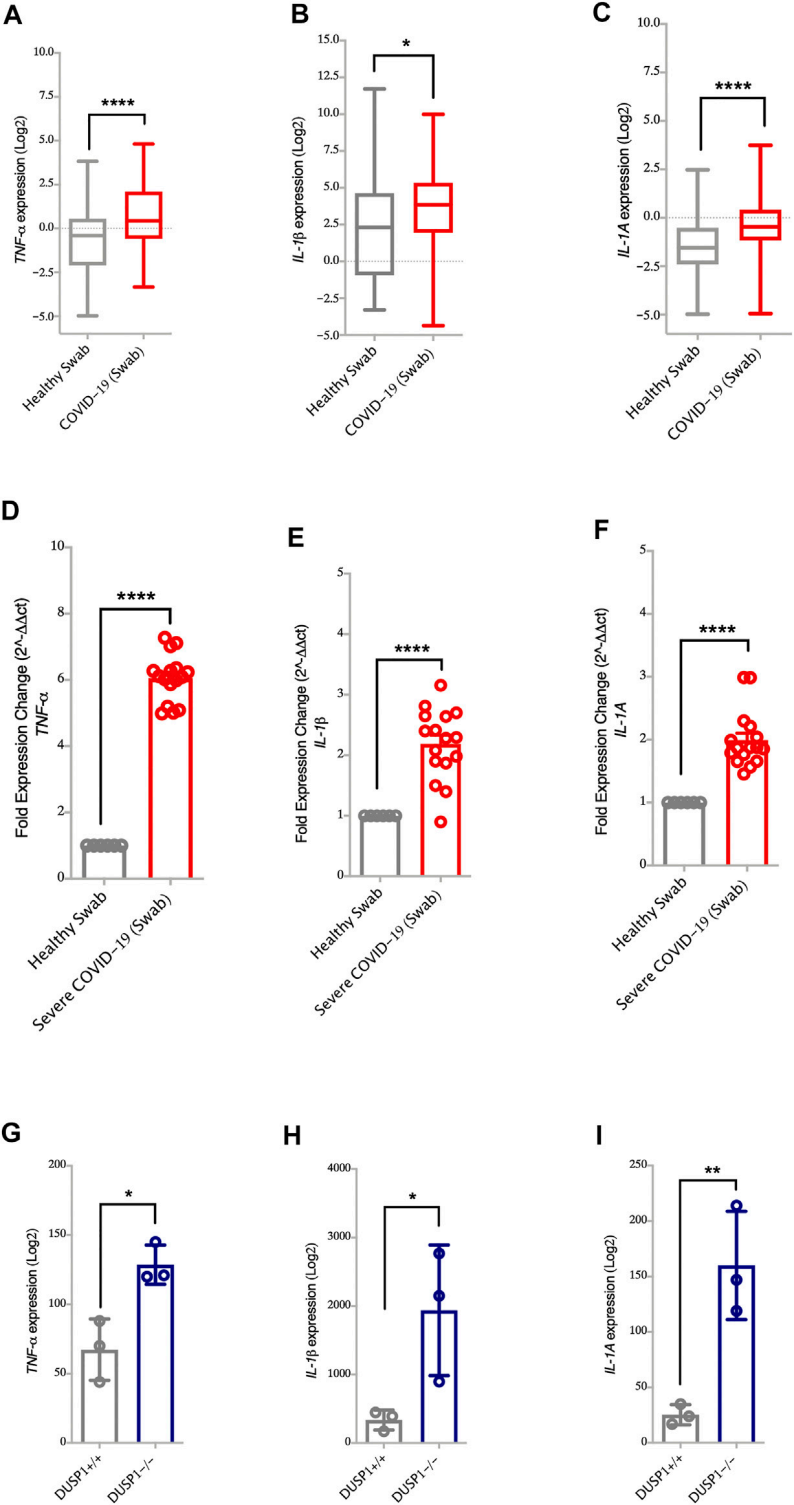
Increased gene expression of MAPK associated pro-inflammatory cytokines in nasopharyngeal swabs of COVID-19 patients **(A–C)** Gene expression level of *TNF-α, IL-1β,* and *IL-1A* in nasopharyngeal swabs fromm COVID-19 (*n* = 430) and healthy controls (*n* = 54) (Dataset GSE152075). Expression level of *TNF-α, IL-1β,* and *IL-1A* is higher in nasopharyngeal swabs of COVID-19 patients as compared to healthy controls **(D–F)** Confirmation of gene expression level of *TNF-α, IL-1β,* and *IL-1A* as measured by RT-PCR, in nasopharyngeal swabs from COVID-19 pateints (*n* = 16) and healthy controls (*n* = 6). Expression level of *TNF-α, IL-1β,* and *IL-1A* is higher in nasopharyngeal swabs from COVID-19 patients as compared to healthy controls **(G–I)** Gene expression level of *TNF-α, IL-1β,* and *IL-1A* in spleen tissue of *DUSP1* ^*+/+*^ mice (*n* = 3) and *DUSP1^−/−^* mice (*n* = 3). Expression level of *TNF-α, IL-1β,* and *IL-1A* is higher in spleen tissue of *DUSP1^−/−^* as compared to *DUSP1* ^*+/+*^ mice. Two-way comparison was done using *t*-test or Mann-Whitney *U* test, depending on the skewness of the data. *p < 0.05, **p < 0.01, ****p < 0.0001.

*DUSP1*, also known as MAPK phosphatase-1 (*MKP-1*), exerts its anti-inflammatory effects through the dephosphorylation of p38 MAPKs. This then reduces the pathway and downregulates the production of *TNF-α, IL-1β*, and *IL-1A* (Jeffrey et al., 2006; Lang et al., 2006). Here, the lower expression of DUSP1 observed in SARS-CoV-2 infected cells and nasopharyngeal swabs of severe COVID-19 patients was associated with the higher expression of *TNF-α, IL-1β*, and *IL-1A* genes (Figures 1A,D and Figures 3A–F, respectively). To further associate *DUSP1* to these cytokines, we have used a data set of *DUSP1* knockout mice (Hammer et al., 2006). Following the LPS challenge, *DUSP1* ^*−/−*^ mice expressed higher mRNA levels of *TNF-α, IL-1β*, and *IL-1A* in the spleen tissue compared to the *DUSP1* ^*+/+*^ mice (Figures 3G–I). This result further highlights the inhibitory role of *DUSP1* on MAPK signaling and pro-inflammatory cytokine production.

### Reduced Gene Expression of DUSP5 Correlated With Enhanced Level of Pro-inflammatory Cytokines

**FIGURE 4:**
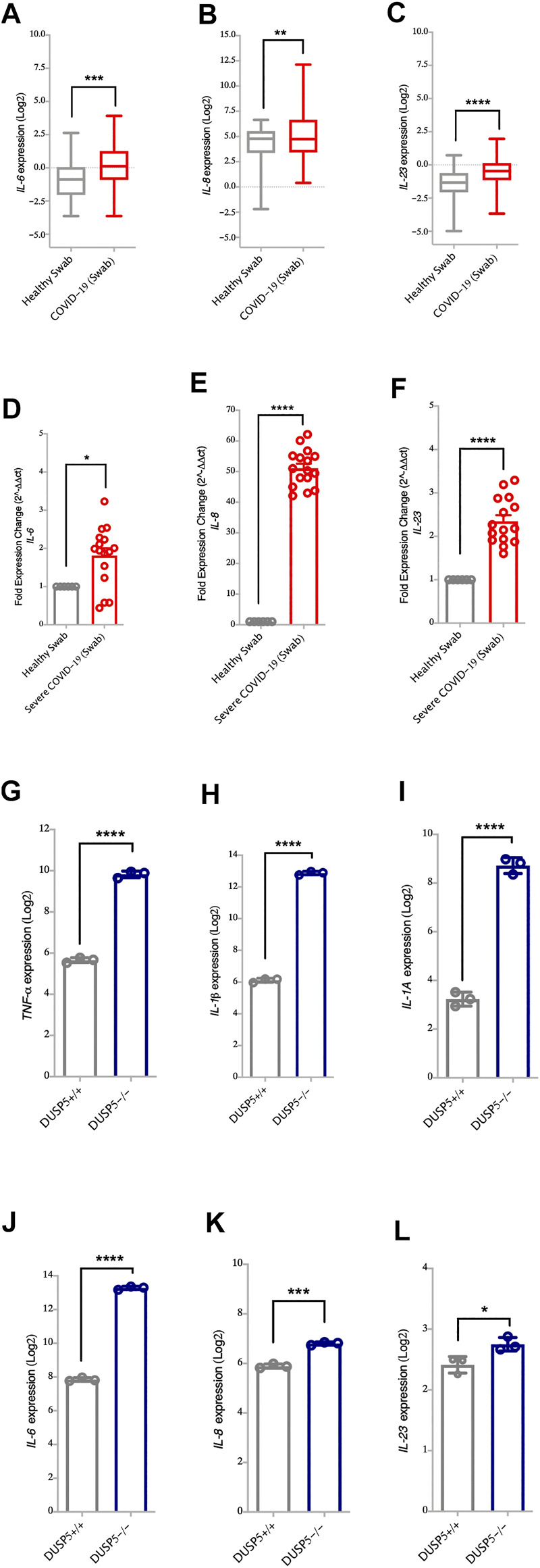
Increased gene expression of NF-κB associated pro-inflammatory cytokines in nasopharyngeal swabs of COVID-19 patients **(A–C)** Gene expression level of *IL-6, IL-8* and *IL-23* in nasopharyngeal swabs from COVID-19 patients (*n* = 430) and healthy controls (*n* = 54) (Dataset GSE152075). Expression level of *IL-6, IL-8* and *IL-23* is higher in nasopharyngeal swabs from COVID-19 patients as compared to healthy controls **(D–F)** Confirmation of gene expression level of *IL-6, IL-8* and *IL-23* as measured by RT-PCR, in nasopharyngeal swabs from COVID-19 patients (*n* = 16) and healthy control (*n* = 6). Expression level of *IL-6, IL-8* and *IL-23* is higher in nasopharyngeal swabs from COVID-19 patients as compared to healthy controls **(G–L)** Gene expression level of *TNF-α, IL-1β, IL-1A, IL-6, IL-23, and IL-8* in bone marrow derived eosinophils from *DUSP5^+/+^* (*n* = 3) and *DUSP5^−/−^* mice (*n* = 3). Expression level of *TNF-α, IL-1β, IL-1A, IL-6, IL-23, and IL-8* was higher in *DUSP5* deficient eosinophils as compared to the WT eosinophils. Two-way comparison was done using *t*-test or Mann-Whitney *U* test, depending on the skewness of the data. *p < 0.05, **p < 0.01, ***p < 0.001, ****p < 0.0001.

It has been reported that DUSP5 functions as a feedback regulator for both p38 MAPKs and NF-κB pathway (Seo et al., 2017). Of note, along with the decreased level of DUSP5, we have observed higher upregulation of MAPKs and NF-κB pro-inflammatory genes such as *TNF-α, IL-1β, IL-1A, IL-6, IL-8* and *IL-23* following SARS-CoV-2 infection as compared to the other coronavirus (Figures 1A,D). The level of these cytokines was also elevated in nasopharyngeal swabs of severe COVID-19 patients compared to healthy controls (Figures 3A–F, for *TNF-α, IL-1β* and *IL-1A*, and Figures 4A–F, for *IL-6, IL-8* and *IL-23*). Moreover, the expression levels of *TNF-α, IL-1β, IL-1A, IL-6, IL-8,* and *IL-23* cytokines were higher in *DUSP5* deficient eosinophils (Holmes et al., 2015), confirming the inhibitory effect of *DUSP5* on MAPKs and NF-κB pathways (Figures 4G–L).

### Chloroquine, Theophylline, and a Range of Anti-inflammatory and Immunomodulatory Medications Increase Expression of DUSP1 and DUSP5

**FIGURE 5:**
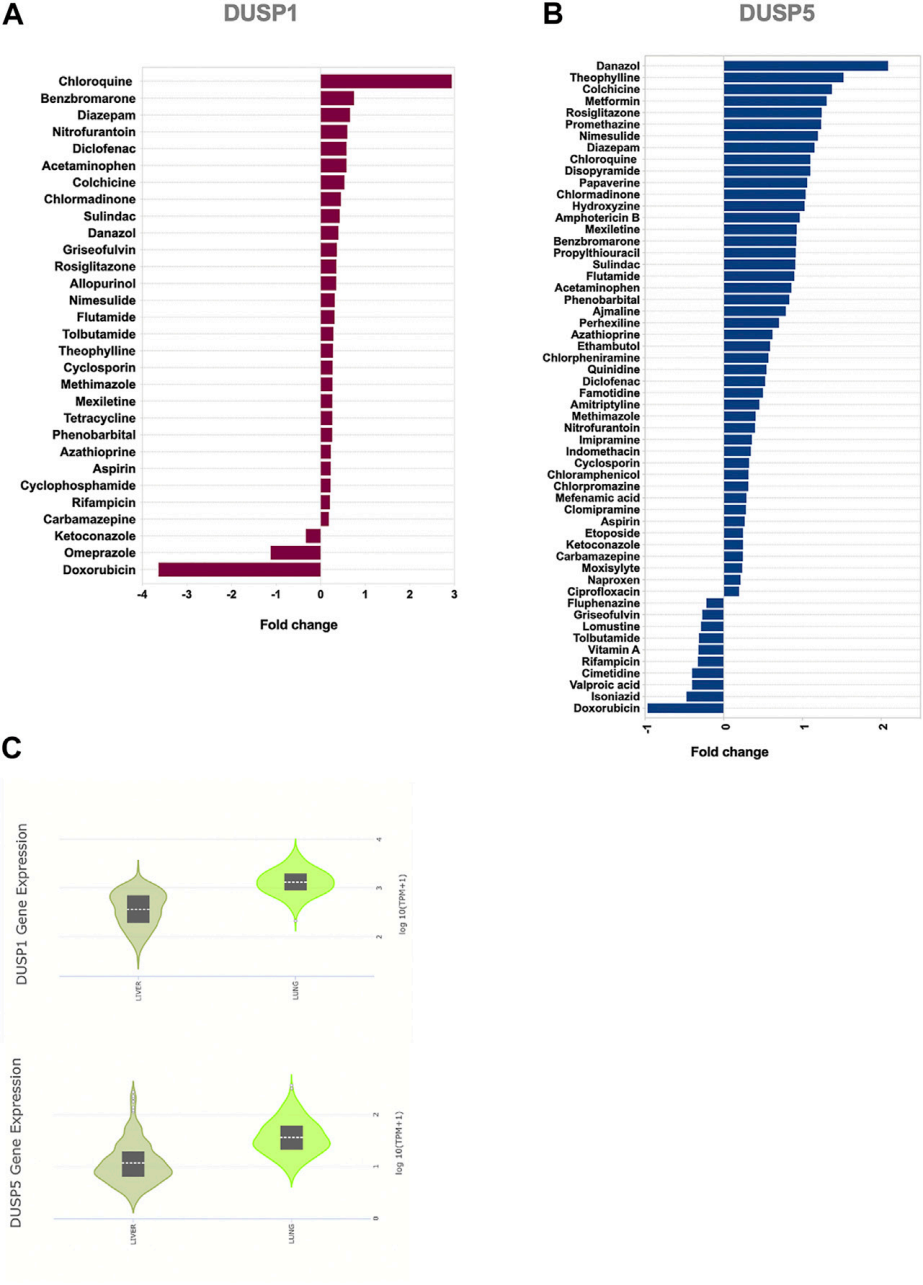
Effect of common medications on the expression levels of *DUSP1* and *DUSP5* in human primary hepatocytes **(A,B)** Medications with a significant regulatory effect on the gene expression levels of *DUSP1* **(A)** and *DUSP5* **(B)**. Statistical test: Limma adjusted *p* < 0.05 **(C)** Baseline expression of *DUSP1* and *DUSP5* in healthy liver and lung tissue extracted from the Genotype-Tissue Expression (GTEx) project. The data shows that the gene expression levels of DUSP1 and DUSP5 in healthy liver tissues, on which the medication has been tested, is comparable with their lung expression levels.

Targeting *DUSP1* and *DUSP5* to modulate MAPK and NF-κB pathways could constitute an attractive approach for the suppression of exaggerated inflammatory responses during COVID-19 infection. We hence used a gene dataset of human primary hepatocytes treated with a wide range of medications. We screened several anti-inflammatory medications for their ability to enhance *DUSP1* and *DUSP5* expression and attenuate MAPKs and NF-κB pathways (Figure 5; Supplementary Table S2). The expression level of *DUSP1* and *DUSP5* was increased noticeably with several medications, such as chloroquine, theophylline, and anti-inflammatory and immune-modulatory medications such as colchicine, diclofenac, cyclosporine, and azathioprine (Figure 5).

Within the analgesics, acetaminophen and non-steroidal anti-inflammatory drugs (NSAIDs) such as diclofenac, sulindac, nimesulide, and aspirin induced an increase in the expression level of *DUSP1* and *DUSP5*. The effect of antihistamines such as hydroxyzine and chlorphenamine was more towards induction of DUSP5 expression.

Another interesting group of medications was anti-diabetics. Rosiglitazone induced an increase in both of *DUSP1* and *DUSP5*; while metformin, a first-line treatment for type 2 diabetes, enhanced *DUSP5* expression (Figure 5; Supplementary Table S2). To our knowledge, the effects of these common medications on the expression of *DUSP1* and *DUSP5* has not been reported before. The ability of these medications to regulate *DUSP1* and *DUSP5* may counteract SARS-CoV-2 modulation of these genes and may hence explain their reported efficiency in controlling inflammation (Seo et al., 2017; Hoppstädter and Ammit, 2019).

## Discussion

In this report, we have shown that, compared to other coronaviruses, SARS-CoV-2 infection downregulates the expression of *DUSP1* and *DUSP5* (leading to MAPK and NF-κB pathways activation) and enhances production of the pro-inflammatory cytokines. MAPKs are fundamental regulators of immune cell function (Dong et al., 2002). In the downstream of the MAPKs are many substrates that are serine/threonine-phosphorylated, which control cytokine mRNA stability and translation. MAPK must be phosphorylated on both threonine and tyrosine residues for kinase activity. The inactivation of MAPK signaling occurs primarily through the dephosphorylation of these substrates motif by members of different MAPK phosphatase (MKP) families, including members of the DUSP family (Chang and Karin, 2001; Jeffrey et al., 2006). Collectively, *DUSP* genes are proposed as molecular brakes of MAPK and NF-κB- modulated inflammation, and hence, they are potential targets for the attenuation of immune responses (Lang et al., 2006).

Of all the DUSP gene family, nuclear *DUSP* genes (*DUSP1, DUSP2, DUSP4*, and *DUSP5*) are the most strongly regulated in activated immune cells (Jeffrey et al., 2006; Lang et al., 2006). Jeffrey et al. (2006) analyzed the regulation of DUSP genes following the activation of B cells, T cells, mast cells, eosinophils, macrophages, and dendritic cells, and found high expression levels of nuclear *DUSPs* in these activated immune cells. Using both *DUSP1* and *DUSP5*-deficient mice datasets (Hammer et al., 2006; Holmes et al., 2015), we also confirmed that knocking out these DUSP genes resulted in the upregulation of MAPK-dependent proinflammatory genes such as *TNF-α, IL-1β* and *IL-1A*.

MAPKs pathways respond to various extracellular stimulations including viral infection, stress, and inflammatory cytokines. Contrary to the SARS-CoV-2 infection, infection with coronavirus infectious bronchitis virus (IBV) resulted in an increase in DUSP1 as viral defense mechanism to reduce p38 MAPK activation and *IL-6* and *IL-8* cytokine production (Liao et al., 2011). Similarly, infections with human respiratory syncytial virus (RSV) and Sendai virus (SeV) upregulated *DUSP1* expression and negatively regulated p38 MAPK (Chang and Karin, 2001).

*DUSP*5 has been shown to inhibit LPS-induced NF-κB signaling (Seo et al., 2017), and its mRNA and protein expression level increased in LPS-stimulated macrophages. *DUSP*5 overexpression in these cells suppressed the production of *TNF-α* and *IL-6*, whereas its knockdown increased their expression (Seo et al., 2017). This was evident here in the context of SAR-CoV-2 infection, as proinflammatory cytokines regulated by MAPK and NF-κB signaling were elevated in the SARS-CoV-2 infected human airway cells compared to infections with other coronaviruses, and in the COVID-19 nasopharyngeal swabs.

The hyperinflammatory response in COVID-19 is characterized by elevated levels of serum *TNF-α, IL-6*, and to a lower level *IL-1A* since this cytokine has a short serum half-life (Del Valle et al., 2020). During COVID-19 infection, pathways upregulated by *TNF-α*, *IL-1β* and type I interferon predominate (Lee et al., 2020). *TNF-α* and *IL-1β* are acute-response cytokines appearing early after infection, followed by a more sustained increase in *IL-6* (Tisoncik et al., 2012). It has been shown that capillary leak, the major factor deteriorating lung function in patients with COVID-19, is driven by *TNF-α*, *IL-1A*, and *IL-6* inflammatory cytokines (Robinson et al., 2020). It is therefore suggested that anti-TNF treatment could reduce inflammation-driven capillary leak in COVID-19; and may reduce the need for mechanical ventilation as well as mortality (Feldmann et al., 2020). Patients with autoimmune diseases on anti-TNF have had reduced serum levels of *TNF-α* and *IL-6* (Ingegnoli et al., 2008). *IL-8,* a chemokine released early after infection*,* plays a key role in the recruitment and activation of neutrophils during inflammatory responses (Baggiolini et al., 1989); and due to the neutrophilia observed in COVID-19 patients, *IL-8* may sustain its pathogenesis (Del Valle et al., 2020). Of note, although *IL-23* has been less reported as a component of the cytokine storm in COVID-19, *IL-23* has a role in the maintenance of Th-17 cells (Di Cesare et al., 2009). Immunomodulatory therapies such as anti-IL-6 have been effective in controlling cytokine storms during COVID-19 and limiting its complications (Xu et al., 2020). Identifying elements contributing to the dysregulation of these pro-inflammatory cytokines during COVID-19, such as reduced expression of *DUSP1* and *DUSP5*, may pave the way for therapeutic intervention to control the level of these cytokines and hence their pathological implications.

Notably, we have shown that treatment with anti-inflammatory agents and analgesics such as acetaminophen and NSAIDs enhance the expression of immune suppresser genes such as *DUSP1* and *DUSP5* resulting in the downregulation of MAPK and NF-κB pro-inflammatory genes. NSAID was found to reduce *IL-6* in the synovial fluid of rheumatoid arthritis patients (Gallelli et al., 2013), and in the sputum of cystic fibrosis patients (Chmiel et al., 2015). Early use of NSAIDs in COVID-19 was suggested to prevent COVID-19 complications (Kelleni, 2021).

Among medications used for COVID-19 (Shehab et al., 2020), chloroquine increased both DUSP1 and *DUSP5* expressions. In an *invitro* study, the anti-inflammatory effect of chloroquine on the inhibition of proinflammatory cytokines such as *TNF-α, IL-1β*, and *IL-6* production from liposaccharide-stimulated macrophages have been reported (Jang et al., 2006). In addition, preliminary clinical trials have supported the efficacy of chloroquine in patients with COVID-19 (Gautret et al., 2020).

Moreover, treatment with theophyline upregulated *DUSP1* expression (Figure 5). Theophylline is a non-selective phosphodiesterase inhibitor and a bronchodilator, used in the treatment of chronic airway inflammatory diseases such as asthma and chronic obstructive pulmonary disease. In combination with glucocorticoids, it restores histone deacetylase activity enhancing the anti-inflammatory effect of steroids (Cosio et al., 2004). Upregulation of *DUSP1* was shown to improve responses to steroid treatment (Newton et al., 2017). This suggests that part of the immune suppressive effect of theophylline is due to its ability to enhance the expression of immune suppressor genes such as DUSP1.

Our results are based on public gene expression datasets and RT-PCR gene expression experiments; thus, they may or may not reflect changes in protein expression. Therefore, confirmatory experiments at the protein levels are needed to support our findings. Given that the SARS-CoV-2 infection does increase the pro-inflammatory cytokines level (Liu et al., 2020), the combined effect of these medications on *DUSP1* and *DUSP5* expression might play a significant role in attenuating the immunopathology of COVID-19. However, more elaborative research is needed to characterize the mechanism through which these medications regulate *DUSPs* expression.

In conclusion, the expression of nuclear *DUSPs*, which are key negative regulators of MAPK and/or NF-κB pathways, are expressed to a lower level during a SARS-CoV-2 infection. Strategies of upregulating these *DUSPs* during a SARS-CoV-2 infection could postulate useful means for manipulating MAPK and NF-κB overactivation; and hence, they may limit the devastating effect of the resulting cytokine storm.

## Data Availability

The datasets presented in this study can be found in online repositories. The names of the repository/repositories and accession numbers can be found below: (NCIB GEO, http://www.ncbi.nlm.nih.gov/geo); (EMBL-EBI, https://www.ebi.ac.uk); and Toxicogenomics Project-Genomics Assisted Toxicity Evaluation System (Open TG-GATEs).

## Abbreviation:

SARS-CoV, Severe acute respiratory syndrome coronavirus.
